# An Overview on *Streptococcus bovis/Streptococcus equinus* Complex Isolates: Identification to the Species/Subspecies Level and Antibiotic Resistance

**DOI:** 10.3390/ijms20030480

**Published:** 2019-01-23

**Authors:** Arianna Pompilio, Giovanni Di Bonaventura, Giovanni Gherardi

**Affiliations:** 1 Department of Medical, Oral and Biotechnological Sciences, “G. d’Annunzio” University of Chieti-Pescara, Via Luigi Polacchi 11, 66100 Chieti, Italy; arianna.pompilio@unich.it (A.P.); gdibonaventura@unich.it (G.D.B.); 2 Center of Excellence on Aging and Translational Medicine, “G. d’Annunzio” University of Chieti-Pescara, Via Luigi Polacchi 11, 66100 Chieti, Italy; 3 Department of Medicine, Campus Biomedico University, Via Alvaro del Portillo 200, 00128 Rome, Italy

**Keywords:** *S. bovis/S. equinus* complex, identification, antibiotic resistance

## Abstract

*Streptococcus bovis*/*Streptococcus equinus* complex (SBSEC), a non-enterococcal group D *Streptococcus* spp. complex, has been described as commensal bacteria in humans and animals, with a fecal carriage rate in humans varying from 5% to over 60%. Among streptococci, SBSEC isolates represent the most antibiotic-resistant species—with variable resistance rates reported for clindamycin, erythromycin, tetracycline, and levofloxacin—and might act as a reservoir of multiple acquired genes. Moreover, reduced susceptibility to penicillin and vancomycin associated with mobile genetic elements have also been detected, although rarely. Since the association of SBSEC bacteremia and colon lesions, infective endocarditis and hepatobiliary diseases has been established, particularly in elderly individuals, an accurate identification of SBSEC isolates to the species and subspecies level, as well as the evaluation of antibiotic resistance, are needed. In this paper, we reviewed the major methods used to identify SBSEC isolates and the antimicrobial resistance rates reported in the scientific literature among SBSEC species.

## 1. Introduction

*Streptococcus bovis/Streptococcus equinus* complex (SBSEC), a non-enterococcal group D *Streptococcus* spp. complex, comprises several species: *Streptococcus equinus*, *Streptococcus infantarius* subsp. *infantarius*, *Streptococcus lutetiensis*, *Streptococcus alactolyticus* and three subspecies of the clade *Streptococcus gallolyticus*, namely *S. gallolyticus* subsp. *gallolyticus* (SGSG), *S. gallolyticus* subsp. *macedonicus* and *S. gallolyticus* subsp. *pasteurianus* (SGSP). SBSEC consists of commensal bacteria, mainly described as colonizers of the rumen, crop, and cloaca of animals and colon of humans, with a fecal carriage rate of SBSEC member in humans ranging from 5% to over 60% [1]. Some SBSEC have been found to cause serious infections such as bacteremia and infective endocarditis in humans, particularly in southern Europe, with the prevalence rising both in animal and elderly patients [2,3,4,5]. The traditional association of SBSEC bacteremia with colorectal cancer was first described in the late 1970s [6] and later extensively confirmed in the literature [7,8,9]. Moreover, an association between *S. bovis* isolation and chronic liver and biliary tract disorders has also been described [10]. Unfortunately, it is still unclear how commensal-to-pathogens transition occurs in SBSEC members, particularly relating to survival, colonization, adhesion, invasion, and interaction with the host immune system. Likewise, the knowledge on the virulence and pathogenicity of SBSEC is limited only to a few adhesion molecules and pro-inflammatory factors [1]. Moreover, members of the SBSEC have also been increasingly identified as important species in the food preparations such as those encountered in food fermentations where they contribute to the quality of the fermented food product [11]. Some SBSEC strains, such as *S. gallolyticus* subsp. *macedonicus* and *S. lutetiensis*, are ingested as part of the daily diet and therefore might be considered to be safe, thus rendering this bacterial group unique among streptococci, containing both pathogenic and “good” strains [1,11].

The difficulties encountered over the years in the correct identification of SBSEC to the species and subspecies level by phenotypic and genotypic methods, made a harmonized analysis of the literature difficult to achieve [12]. This situation is further complicated by the lack of an optimal molecular method for the correct identification to the species level. Moreover, the current classification system remains subject to debate and is not uniformly accepted due to the absence of a curated sequencing database, the lack of revised nomenclature in culture collection deposits, and the imperfect updates to commercial phenotypic identification database systems. The introduction of the new nomenclature of SBSEC species and subspecies revealed specific diseases associations among the different species. SGSG represents the major cause of infective endocarditis and monomicrobial bacteremia, associated with colorectal cancer [13,14,15]. In particular, SGSG has been demonstrated to carry unique virulence factors facilitating both the infection through premalignant colonic lesions and the innate immune system evasion, and the formation of biofilm at collagen-rich sites in susceptible patients with colorectal cancer [14]. SGSP and *S. infantarius* seem, instead, mainly related to immunosuppressive comorbidities and polymicrobial bacteremia, while being associated with biliary-pancreatic diseases and biliary tract infections. *S. infantarius* is most frequently associated with bile duct and biliary-pancreatic cancers, whereas SGSP is associated with benign biliary tract diseases [16,17] and, at lesser extent, to urinary tract infections, osteoarticular infections, gastrointestinal infections, and meningitis, mostly in elderly patients [18,19,20,21,22,23]. Geographic differences in epidemiology and prevalence occur among SBSEC species: SGSG is the most frequent species causing infective endocarditis in Europe, whereas SGSP seems to be more prevalent in Asia [24].

Among streptococci, SBSEC isolates represent the most antibiotic-resistant species, with variable resistance rates observed for clindamycin, erythromycin, tetracycline and levofloxacin [11]. Even though penicillin-resistant isolates have still not been observed, reduced susceptibility to penicillin has been rarely reported [25]; moreover, vancomycin resistance genes being carried on mobile genetic elements due to horizontal gene transfer from commensal fecal microbiota have also been detected, although rarely.

The present narrative review is focused on the major diagnostic methods used to identify SBSEC isolates, as well as on the antimicrobial resistance rates reported among SBSEC species.

## 2. Diagnostic Methods for the Identification of SBSEC

To retrieve published works on diagnostic methods for SBSEC identification, we searched PubMed electronic database for all eligible studies focused on identification of *S. bovis* and/or *S. gallolyticus* isolates published since 2000, as well as the more cited papers. The search was restricted to all papers that clearly reported data on identification of isolates belonging to SBSEC and those in the English language only. The results of diagnostic methods reported for the identification of SBSEC are summarized in Table 1.

The identity of SBSEC strains in human diseases has not been systematically investigated using modern taxonomy. Considering the specific association between diseases and microbiology features, accurate identification of SBSEC isolates is mandatory. The massive changes in the SBSEC taxonomy have resulted in confusing use of SBSEC species’ names in the scientific literature due to many studies published before the recommended current taxonomy, and to the evidence that the current taxonomy has not been completely adopted. 

Traditionally, SBSEC has been classified into the non-enterococcal group D Lancefield antigen *Streptococcus*, and the identification to the bacterial species was initially performed by phenotypic tests [26]. In the early 1960s, the development of classification systems placed *S. bovis* into a more defined scheme among the Group D streptococci. The main biochemical differences between group D enterococci and *S. bovis* were based on the ability to grow in 6.5% NaCl, hydrolyze arginine, and decarboxylate tyrosine [27]. Due to the increased importance of SBSEC isolates in human infections, Facklam emphasized the importance of an accurate speciation by phenotypic methods to understand the species distribution of group D streptococci among human infections, as well as antimicrobial susceptibilities [28]. Based on phenotypic/biochemical tests, SBSEC species were grouped into 2 biotypes: biotype I (mannitol-fermenting) and biotype II (mannitol-nonfermenting). Biotype II was subdivided into II/1 and II/2, based on trehalose fermentation, β-galactosidase and β-glucuronidase activities, and starch degradation [17,28,29]. Phenotypic biochemical methods have been the most common species identification methodologies used in routine diagnostic laboratories in recent years, though they have limited differentiation capacity due to the phenotypic variability [30,31,32,33,34,35,36]. Moreover, they are time-consuming techniques providing species identification only after 24–48 h. For full biochemical and phenotypic descriptions, we invite to refer to Bergey’s Manual and their implementations in API and VITEK identification approaches [37].

Using the scheme proposed by Schlegel et al. [12] based on DNA studies, SBSEC taxonomy significantly changed, and later significantly improved using methodologies based on DNA-DNA hybridization, and single-gene-based molecular testing (i.e., 16S rRNA, *groEL*, and *sodA*). This allowed us to reclassify SBSEC into 7 different (sub)species grouped into four clusters, with two *Streptococcus* species of principal interest in human pathogenesis: *S. gallolyticus* - with the subspecies SGSG, formerly biotype I, and SGSP, formerly biotype II/2 - and *S. infantarius*, formerly biotype II/1, with the subspecies *coli* and *infantarius* [12,17,38,39]. Partial sequence comparison of *rpoB*, *sodA*, *groEL*, and *gyrB* within the genus *Streptococcus* indicated that these genes are more discriminative than 16S rRNA gene sequence, and partial *groEL* gene sequence comparison generally represents the best tool for the identification at species and subspecies levels [40].

Besides the phenotypic-based approaches, diagnostic methods used for identification of SBSEC also include proteomic-based MALDI-TOF (Matrix Assisted Laser Desorption Ionization Time-of-Flight analysis), and genomic-based methods, such as single-gene-based molecular testing (16S rRNA, *sodA*, *groEL*, and other genes), 65 and the recently introduced whole genome sequencing. Multilocus sequence typing has also been successfully applied to identify and discriminate species of the SBSEC [5,41,42]. It has been clearly demonstrated that no single test system among phenotypic, molecular or proteomic methods can provide unequivocal identification, whereas a combination of these techniques is often used to achieve the best performance for the accurate identification of SBSEC to the species level.

Several molecular methods have been developed to improve species identification of streptococci, included SBSEC, such as PCR and sequencing of 16S rRNA, *rnpB*, *groEL*, and *sodA*, with different outcomes [22,43,44].

Partial or complete nucleotide sequences of 16S rRNA amplicons have been found to be useful for SBSEC identification [16,45]. It has been reported that the detection of four different point mutations in 16S rRNA could be essential to discriminate among SGSG, SGSP and *S. infantarius* subsp. *coli* [39]. We recently found that 16S rRNA gene sequencing successfully clusters SGSG and SGSP into two separated and well-defined groups with the use of the phylogenetic tree provided by a curated website [46]. A study on 172 bacteremic *S. bovis* complex isolates - comprising SGSG, SGSP and *S. infantarius*—confirmed subspecies identification by sequencing of both 16S rRNA and *sodA* genes and by PCR-RFLP (Restriction-Fragment-Length-Polymorphism) assays of *groESL* gene [43]. PCR-RFLP assay based on *groESL* sequences combined with Vitek2 was also used to study 24 bacteremic cases with SBSEC infections, encompassing 13 SGSP, six SGSG, four *S. infantarius* subsp. *coli*, and one *S. infantarius* subsp. *infantarius* [47]. A multiplex PCR assay comprising the 16S rRNA gene followed by RFLP has been successfully applied on 200 SBSEC isolates from dairy products and reference strains, thus representing another molecular method developed to improve species identification of SBSEC able to discriminate *Streptococcus infantarius* (biotype II.1) from *Streptococcus gallolyticus* (biotype I and II.2)/*Streptococcus alactolyticus* and *S. equinus* [48]. In several cases, partial *sodA* gene sequencing was more discriminant than the 16S rRNA [33,36,49,50]. Moreover, partial sequencing of ribosomal protein S2 gene, *rpsB*, was successfully applied to cluster SGSG and SGSP isolates responsible for cases of meningitis in adults [21].

Real-time PCR assays based on the *recN* and *gyrB* genes have been developed to reliably detect from rectal swab specimens SBSEC subspecies, namely SGSG, SGSP, *Streptococcus infantarius* subsp. *coli*, and *S. infantarius* [51]. Moreover, DNA sequencing of the 16S–23S intergenic spacer (ITS) region has also been used to retrospectively identify SBSEC isolates recovered from blood cultures [23]. Recently the use of amplification and sequencing of *tanB* for SGSG, and of the SGPB0680 cell wall surface protein gene for SGSP was demonstrated to be accurate in the identification of six clinically relevant streptococcal species, including SBSEC isolates [52].

Recently, the MALDI-TOF MS (Mass-Spectrometry) technique has gained considerable interest in many microbiology laboratories where it is currently the main method for species identification. It is a very fast and cheap methodology showing similar and, in some cases, better performance than 16S rRNA gene sequencing [53,54]. Nevertheless, in some cases, the accuracy of the MALDI-TOF methodology is limited to the identification of a specific bacterial complex or group, as it is also used for some streptococcal species, including those of SBSEC. A limitation of MALDI-TOF Bruker in discriminating SBSEC species has been reported with variable performance and high dependence on the system, spectral databases and algorithms used [19]. Recently, the comparative evaluation of two MALDI-TOF systems, Bruker Biotyper and Vitek MS, considering 16S rRNA and 16S-23S intergenic spacer region sequencing as the reference method, revealed several inaccuracies, therefore suggesting the need for additional optimization of the available system databases or identification algorithms [55]. Conversely, other works reported the usefulness of MALDI-TOF to identify streptococcal species correctly. Hinse et al. demonstrated a good SBSEC species- and subspecies-level of discrimination based on dendrogram analysis of mass spectral profiles using the SARAMIS database (bioMérieux Italia, Florence, Italy) with MALDI-TOF MS instrument (Shimadzu Corp., Kyoto, Japan), using *sodA* gene sequencing as the reference method [56]. Similarly, another study that also used *sodA* gene sequencing as the gold standard method confirmed the usefulness of MALDI-TOF Bruker technology to properly discriminate between SGSG and SGSP, with some problems encountered with *S. equinus* species [57]. Moreover, another report on a collection of 54 and 97 streptococcal type strains and clinical isolates, respectively, including SBSEC species, revealed both MALDI Biotyper (Bruker Daltonics Italia, Macerata, Italy) and VITEK-MS (bioMérieux) to be reliable and accurate in clinical diagnostics for streptococcal identification compared with 16S rRNA gene sequencing as the reference method, providing better results than commercial biochemical methods [58]. Another comparative study on the identification of bacteremic streptococcal species, comprising SBSEC and based on *rnpB* gene sequencing and 2 MALDI-TOF systems, MALDI Biotyper (Bruker) and VITEK MS IVD (bioMérieux), showed excellent and comparable performances of the three methods [44]. Finally, in a recent study, we confirmed the usefulness of MALDI Biotyper (Bruker) and Vitek MS (bioMérieux) systems to identify SGSP isolates to the species level correctly, although only Bruker Biotyper accurately identified strains to the subspecies level [46].

## 3. Antimicrobial Resistance

The widespread use of antibiotics caused a relevant selective environmental pressure, selecting antibiotic-resistant bacterial species and favoring their spread. This selection reflects the increasing presence of antibiotic-resistant commensal bacteria in the gut microbiota of both humans and animals that might act as a reservoir of antibiotic resistance genes with the potential of being transmitted to pathogens through genomic exchange. Among them, enterococci are the most abundant genus found in the human gastrointestinal tract [59], but streptococci are also represented, mainly by SBSEC isolates. 

In this narrative review, we included all papers published since 2000 that reported resistance rates among SBSEC isolates. Papers in languages other than English, review articles, papers that included a small number (less than 15) of SBSEC isolates, and those where the number of isolates or antibiotic resistance rates was not clearly reported, were all excluded from this analysis. 

Based on these criteria 16 articles were overall retrieved, and the results of antibiotic resistance rates are summarized in Table 2. Overall, tetracycline, erythromycin, and clindamycin were the antimicrobial agents showing the highest resistance rates, ranging between 36% and 77% for tetracycline, 8.9% and 78% for erythromycin, and between 10.6% and 62% for clindamycin. The most recent study retrieved was from Italy and performed on SGSP isolates collected between 2010 and 2012: 31.8% were erythromycin-resistant and positive for *erm*(B) resistance gene, whereas all of them were also clindamycin-resistant [46]. Moreover, 68.2% of SGSP strains were tetracycline-resistant, most of those carrying *tet*(O), whereas a minority harbored the *tet*(M) gene [46]. A previous study from Italy was conducted on 25 *S. bovis* isolates responsible for endocarditis or bacteremia during 1990–2003 and classified as SGSG (20 isolates), SGSP (4 isolates), and *S. infantarius* (SI, 1 isolate). All isolates were susceptible to penicillin, glycopeptides, and linezolid, whereas 4%, 48%, 8%, and 64% of isolates were resistant to levofloxacin, erythromycin, gentamicin, and tetracycline, respectively [60]. Among 45 *S. bovis* isolates causing bacteremia recovered from 2003 to 2010 in Spain and re-identified according to the new taxonomic scheme, all isolates remained susceptible to penicillin, ampicillin, amoxicillin-clavulanate, oxacillin, quinupristin-dalfopristin, linezolid, and rifampin, whereas resistance rates of 33.3%, 15.6%, and 20.2% were reported, respectively for erythromycin, levofloxacin, and co-trimoxazole [19]. Variable resistance rates according to the bacterial species were reported and *S. lutetiensis* showed the highest resistance rates for erythromycin and clindamycin (60% for both) [19]. Lower erythromycin resistance rates (less than 20%) were reported in two separate studies. Among 45 independent carriage *S. bovis* isolates from Israel, approximately 9% of isolates were erythromycin-resistant [61]. In Japan, all 66 *S. gallolyticus* isolates recovered from various sources in both humans and animals between 1981 and 2011 were susceptible to vancomycin, penicillin G, and ampicillin. Low resistance rates were found for erythromycin, clindamycin, cefotaxime, and chloramphenicol (16.7%, 10.6%, 15.2%, and 4.5%, respectively) with the majority of erythromycin-resistant isolates harboring *erm*(B) gene [62]. Higher resistance rates were observed for tetracyclines (56.6% and 68.2%, respectively for tetracycline and doxycycline), with all tetracycline-resistant isolates showing either *tet*(M), *tet*(O), or *tet*(L) [62].

Erythromycin resistance rates higher than 40% were found in other studies. Assessing the activity of daptomycin against bacteremic streptococci from an international collection including 100 *S. bovis* isolates, Streit et al. [63] found that all *S. bovis* isolates were susceptible to vancomycin, daptomycin, and linezolid, whereas 97% and 94% were susceptible respectively to penicillin and quinupristin/dalfopristin; resistance rates for erythromycin, clindamycin, and tetracycline were of 46%, 26%, and 65%, respectively. In Hong Kong, China, among 48 SBSEC bacteremic isolates (mainly SGSP) collected over the period 1996–2001, an erythromycin resistance rate of 65% was found, mostly associated with *erm*(B) and *erm*(T), and a clindamycin resistance rate of 41% [24]. Another study from China, investigating clinical and microbiological features of 23 episodes of peritoneal dialysis peritonitis caused by *S. bovis*, reported resistance rates to clindamycin and erythromycin of 43.5% and 47.8%, respectively, [64]. A recent study from Taiwan, among 172 SBSEC collected between 2000 and 2012—including SGSG (126 isolates), SGSP (31 isolates), and *S. infantarius* (15 isolates)—54.7% of isolates were erythromycin-resistant, mostly showing the iMLS_B_ phenotype (inducible resistance to macrolide, lincosamide, and streptogramin B antibiotics), thus with concomitant resistance also to clindamycin (54.1%) [43]. *S. infantarius* was the most frequent SBSEC species associated with multidrug-resistance [43]. In another previous study from Taiwan performed on 60 SBSEC blood isolates, mostly SGSP, collected between 1996 and 2000, a total of 63.3% of erythromycin-resistant strains were identified associated with the resistance genes *erm*(B) or *erm*(T) [65].

Leclercq et al. reported 59.4% of macrolide resistance rate in 128 clinical SBSEC isolates, mainly belonging to SGSG, with the vast majority harboring the resistance gene *erm*(B) [66]. Overall, 77.7% of isolates were tetracycline-resistant associated with *tet*(M), *tet*(L), and/or *tet*(O) [66]. Several studies from Spain conducted on SBSEC and complying the criteria used in the present review reported variable antibiotic resistance rates for erythromycin (48-78%) and clindamycin (45–72%). Matesanz et al. studying 118 SBSEC isolates, mostly SGSP, causing bacteriuria in adult patients and recovered during the period 2003–2012, reported that all isolates were susceptible to penicillin, whereas 48% and 45% of strains were resistant to erythromycin and clindamycin, respectively [20]. In a study on 41 SBSEC isolates from animals and humans collected between 1990 and 2010, 46.3% of isolates were erythromycin-resistant all but one carrying *erm*(B), 48.8% were clindamycin-resistant, and 36.6% were minocycline-resistant isolates all harboring *tet*(M) [67]. Indeed, 5 isolates were gentamycin-resistant with *aac*(6’)-*aph*(2’) gene, 6 (14.6%) were quinupristin-dalfopristin resistant, 5 (12.2%) were levofloxacin-resistant, and 3 (7.3%) were cotrimoxazole-resistant [67]. Another study from Spain conducted among 107 consecutive cases of bacteremia with or without infective endocarditis caused by SBSEC, prevalently SGSG, over two periods (1988–1996 and 1997–2005), reported that all isolates were penicillin-susceptible, whereas resistance rates for erythromycin, clindamycin, and cotrimoxazole were 60.2%, 54.5%, and 85.3%, respectively [68]. In a previous prospective study focused on 64 isolates belonging to SBSEC species and causing significant bacteremia in adult patients during the period 1987–2003, the totality of the isolates was susceptible to penicillin, cefotaxime, and vancomycin, whereas 60%, 60%, and 50% were resistant to erythromycin, trimethoprim/sulfamethoxazole, or clindamycin, respectively [69]. Finally, in another in vitro study aimed at evaluating the susceptibility profiles of 18 *S. bovis* bloodborne isolates collected between 1998 and 2003, 11% of isolates showed reduced susceptibility to penicillin whereas 78%, 72%, and 39% were resistant to erythromycin, clindamycin, or telithromycin, respectively [25]. All but one erythromycin-resistant isolate carried *erm*(B), whereas the remaining isolate carried *mef*(A) [25]. 

Regarding the mechanisms underlying the macrolide resistance, *erm*(B) has been identified as the most frequent erythromycin-resistant determinant, followed by *erm*(T) and *mef*(A) [24,25,46,62,65,66,67]. Moreover, *erm*(T) gene—identified flanking the mobile element IS*1216V*—has also been described in 6 inducible erythromycin-resistant SGSP isolates [70]. Recently, 6 SGSP isolates from dead ducklings collected in China during 2010–2013 were found to exhibit multi-drug resistance, including high macrolide resistance and carried *erm*(B) and *erm*(T) genes, clustering with Tn916 and IS1216, respectively [71]. Tetracycline resistance has been described to be mainly associated with *tet*(M), followed by *tet*(O). Moreover, a recent molecular characterization of 39 foodborne tetracycline-resistant isolates of *S. bovis* identified a novel mosaic *tet*(S/M) fusion [72]. Indeed, a new mechanism has been recently identified associated with macrolide resistance: two previously unknown conjugative transposons, Tn6263 and Tn6331, were found to confer aminoglycoside/macrolide co-resistance identified in a clinical isolate of SGSG responsible for infective endocarditis and colorectal cancer [73].

Several works reported reduced susceptibility to penicillin among SBSEC bacterial species. Although very low rates of reduced susceptibility to penicillin have been reported in some studies, ranging between 0.6% to 3% [43,63], other papers reported higher rates of low levels of penicillin-resistance (Table 2). Thirty percent of isolates from China causing peritoneal dialysis peritonitis showed moderate resistance to penicillin [64]. Reduced susceptibility to penicillin (MIC: 0.75 mg/L) was also reported in a clinical isolate of *S. bovis* biotype II/2 (SGSP) causing neonatal meningitis [74]. Among carriage *S. bovis* isolates from Israel, approximately 13% of isolates were penicillin-resistant [61]. Similarly, a reduced susceptibility to penicillin among *S. bovis* isolates with a rate of 11% has been reported by Rodrıguez-Avial et al. [25] (Table 2).

Vancomycin resistance has been rarely reported in SBSEC isolates from animal fecal samples and has been mainly associated with *vanB* gene carried on transferable elements. The finding of a vancomycin-resistant strain from a stool swab in human is of concern [67,75,76,77]. Comparative genomic analyses revealed that such a strain underwent extensive genetic elements exchange and chromosomal rearrangements with the acquisition of an unusually high number of transposable elements [67] (Table 2). With regard to the other antimicrobial agents, it is worthwhile to mention the high resistance rates for trimethoprim-sulfamethoxazole reported in some studies, varying from 7% to 98% [19,20,67,68,69].

## 4. Conclusions

The identification to the species level of SBSEC is mandatory because of the specific disease association reported in the literature. The combination of proteomic and molecular methods allows the correct and precise identification of SBSEC to the species level, and the simultaneous application of multiple identification methods along with the clinical presentation of the patient seems to be essential. However, whole genome studies will be useful in the future to improve the accuracy of identification and for identifying specific virulence factors associated with specific diseases.

SBSEC isolates, along with other gut *Firmicutes* such as enterococci and eubacteria, are resident of the gastrointestinal tract and may represent potential reservoirs for horizontal gene transfer of virulence factors and antibiotic resistance genes among the mammalian gastrointestinal microbiota. This, added to the evidence that antibiotic resistance is widespread among the SBSEC clinical isolates, representing a serious problem due to the increasing infection rates, makes necessary the continuous monitoring of resistance profiles in SBSEC isolates.

## Figures and Tables

**Table 1:** Identification methods reported in the literature for SBSEC isolates.

Identification Method	*S. alactolyticus*	*S. equinus*	*S. gallolyticus* subsp. *gallolyticus*	*S. gallolyticus* subsp. *macedonicus*	*S. gallolyticus* subsp. *pasteurianus*	*S. infantarius* subsp. *infantarius*	*S. lutetiensis* (*S. infantarius* subsp. *coli*)	Reference	Comments
**Phenotypic**									Rarely used. Lack of revised nomenclature in culture collection deposits and imperfect updates of databases.
Rapid ID 32 Strep (bioMérieux)	+/-	-	-	-	-	-	-	[17,33,39]	
Vitek 2 GP ID Card (bioMérieux)	+/-	+/-	+/-	-	+/-	+/-	+/-	[14,17,21,22,43,47]	
**Genotypic ^a^**									Generally based on gene PCR and sequencing. Partial sequences of *rpoB*, *sodA*, *groEL*, and *gyrB* are more discriminative than 16S rRNA gene sequence, with *groEL* representing the best performer. Absence of curated sequencing databases and lack of revised nomenclature in culture collection strains.
16S rRNA	n.a.	+	+	+	+	+/-	+/-	[14,19,22,39,43,45,46,48,58]	
*soda*	+	+	+	+	+	+	+	[19,22,33,40,43,56,57]	
*rpsB*	n.a.	n.a.	+	n.a.	+	n.a.	n.a.	[21]	
*gyrB*	+	+	+	+	+	+	+	[40,51]	
16S-23S ITS R	n.a.	+	+	+	+	+	+	[23,55]	
*tanB*	n.a.	n.a.	+	n.a.	n.a.	n.a.	n.a.	[52]	
SGPB0680	n.a.	n.a.	n.a.	n.a.	+	n.a.	n.a.	[52]	
*rpoB*	+	+	+	+	+	+	+	[40]	
*groES/EL* ^b^	+	+	+	+	+	+	+	[40,43,47]	
*rnpB*	n.a.	n.a.	+	n.a.	+	n.a.	n.a.	[44]	
*recN* ^c^	n.a.	n.a.	+	n.a.	+	+	+	[51]	
MLST ^d^	+	+	+	+	+	+	+	[5,42]	
**Proteomic**									Very fast and cheap, but highly dependent on the system, spectral databases and algorithms used
MALDI TOF Bruker Biotyper	n.a.	-	-	-	+/-	-	+	[19,44,45,46,55,57,58]	
MALDI TOF Vitek MS	+	+	+	+/-	+/-	+	+	[44,45,46,55,56,58]	

From the literature a given method has been reported to: correctly identify SBSEC isolates to the species/subspecies level with high probability (“+”); be not able to correctly identify isolates to the species/subspecies level or correctly identify isolates with low probability (“-“); show discordant results (“+/-”); “n.a.” indicates that the SBSEC species/subspecies was not tested by the corresponding method. ITS R: interspacer region. ^a^ Genotypic methods are mainly based on gene PCR and sequencing. For 16S rRNA gene restriction fragment length polymorphism (RFLP) analysis has been also reported [48]. For *gyrB* gene real-time PCR without sequencing has been also described [51]. Refer to the text for details. ^b^ RFLP analysis of *groESL* gene has been also described [43,47]. ^c^ Real-time PCR for *recN* gene has been described [51]. ^d^ Multilocus Sequence Typing (MLST) based on PCR and sequencing of 7 housekeeping genes (*dpr*, *gmk*, *rpoD*, *parC*, *pta*, *pyrC*, *recN*) [42] or 10 housekeeping loci (*ddlA*, *gki*, *glnA*, *mutS*, *mutS2*, *pheS*, *proS*, *pyrE*, *thrS*, *tpiA*) [5].

**Table 2:** Antibiotic resistance rates and resistance genes reported in the literature regarding species belonging to the *Streptococcus bovis/Streptococcus equinus* complex (SBSEC).

Isolates (*n*)	Origin (Human or Animal)	Body Site	Study Period	SBSEC Species/ Subspecies (*n*) ^a^	Ery-R (%)	Ery-R Gene (%)	Cli-R (%)	Tetracyclines-R (%) ^b^	Tet-R Gene ^c^	Other Antibiotics (%) ^d^	Country	Ref.
22	Human	Various	2010–2012	SGSP (22)	31.8	*erm*(B) (100)	31.8	68.2	*tet*(O) *tet*(M)	Pen (0) Ctx (0) Van (0)	Italy	[46]
25	Human	Blood	1990–2003	SGSG (20) SGSP (4) SI (1)	48	n.a.^h^	n.a.	64	n.a.	Gen (8) Lev (4) Pen (0) Van (0) in (0)	Italy	[60]
45	Human	Blood	2003–2010	SGSG (14) SGSP (24) SISI (2) SL (5)	33.3	n.d.	30.2	n.a.	n.a.	Sxt (20.2) Lev (15.6) Pen (0) Str (0) Rif (0) Li (0) Q-D (0)	Spain	[19]
100	Human	Blood	n.a.	*S. bovis*	46	n.a.	26	65	n.a.	Pen (3) Van (0) Lin (0) Dap (0) Q-D (6)	International collection	[63]
45	Human	Carriage	n.a.	*S. bovis*	8.9	n.a.	n.d.	n.a.	n.a.	Pen (13.3) Cro (0) Van (0)	Israel	[61]
48	Human	Blood	1996–2001	SGSG (2) SGSP (42) SI (4)	65	*erm*(B) (54.2) *erm*(T) (41.7) *erm*(B)+*erm*(T) (4.2)	41	n.a.	n.a.	Pen (0) Van (0)	China	[24]
23	Human	Peritoneal dialysis	2000–2010	*S. bovis*	47.8	n.a.	43.5	n.a.	n.a.	Pen (30.4)	China	[64]
172	Human	Blood	2000–2012	SGSG (126) SGSP (31) SI (15)	54.7	n.a.	54.1	n.a.	n.a.	Gen (19.8) Lev (16.9) Pen (0.6) Cro (0) Van (0) Lin (0) Dap (0) Tig (0)	Taiwan	[43]
60	Human	Blood	1996–2000	SGSG (4) SGSP (53) SI (3)	63.3	*erm*(B) (63.2) *erm*(T) (36.8)	63.3	75	*tet*(M) ^e^	Pen (0) Ctx (0) Van (0) Chl (0)	Taiwan	[65]
66	Human (20) Animal (44) Reference (2)	Various	1981–2011	SG	16.7	*erm*(B) (63.7)*mef*(A) (9.1) *erm*(B)+*mef*(A) (9.1) no genes (18.2)	10.6	57.6	*tet*(M) *tet*(O) *tet*(L) *tet*(T)	Ctx:15.2 Chl:4.5 Pen:0 an:0	Japan	[62]
128	Human (125) Animal (3)	Blood or cardiac biopsy	1994–2003	SGSG (121) SGSP (3) SI (2) SL (2)	59.4	*erm*(B) (96.1) *erm*(B)+*mef*(A) (2.6) *mef*(A) (1.3)	58.6	77.7	*tet*(M) *tet*(M)+*tet*(L)+*tet*(O) *tet*(M)+*tet*(O) *tet*(M)+*tet*(L) *tet*(O) *tet*(L)	Pen (0) Van (0) Gen (0) Rif (0)	France Belgium Netherlands	[66]
118	Human	Urine	2003–2012	SGSG (15) SGSP (85) SI (18)	48	n.a.	45	75 ^f^	n.a.	Sxt (98) Fos (23) Lev (16) Nit (2) Pen (0) Ctx (0)	Spain	[20]
41	Human (18) Animal (23)	Various	1990–2010	SGSG	46.3	*erm*(B) (94.7) o genes (5.1)	48.8	36.6 ^g^	*tet*(M)	Fos (51.2) Q-D (14.6) Gen (12.2) Lev (12.2) Sxt (7.3) Van (2.4)	Spain	[67]
107	Human	Blood	1988–2005	SGSG (69) SGSP (38)	60.2	n.a.	54.5	n.a.	n.a.	Sxt (85.3) Pen (0)	Spain	[68]
64	Human	Blood	1987–2003	SGSG (42) SGSP (22)	60	n.a.	50	n.a.	n.a.	Sxt (60) Pen (0) Ctx (0) Van (0)	Spain	[69]
18	Human	Blood	1998–2003	*S. bovis*	78	*erm*(B) (92.9) *mef*(A) (7.1)	72	n.a.	n.a.	Tel (39) Pen (11)	Spain	[25]

Abbreviations: SG: *Streptococcus gallolyticus*; SGSG: *S. gallolyticus* subsp. *gallolyticus*; SGSP: *S. gallolyticus* subsp. *pasteurianus*; SI: *Streptococcus infantarius*; SISI: *Streptococcus infantarius* subsp. *infantarius*; SL: *Streptococcus lutetiensis*; Pen: Penicillin; Ctx: Cefotaxime; Cro: Ceftriaxone; Van: Vancomycin; Gen: Gentamycin; Lev: Levofloxacin; Lin: Linezolid; Sxt: Trimethoprim/Sulfamethoxazole; Str: Streptomycin; Rif: Rifampicin; Q-D: Quinupristin-Dalfopristin; Dap: Daptomycin; Tig: Tigecycline; Chl: Chloramphenicol; Fos: Fosfomycin; Nit: Nitrofurantoin; Tel: Telithromycin. ^a^ *S. bovis* indicates that the species/subspecies were not reported in that study. ^b^ Tetracyclines refer mainly to tetracycline. When others are tested, i.e., doxycycline or minocycline, these are indicated (see footnotes “f” and “g”). ^c^ Tetracycline resistance genes are listed in order of frequency reported in each study, and relative percentages are not indicated. ^d^ Resistance rates to other antibiotics tested in each study, excluded erythromycin, clindamycin, and tetracyclines. Isolates with reduced susceptibility to penicillin and cephalosporins, such as cefotaxime and ceftriaxone, are also included. ^e^ The presence of tetracycline resistance genes is evaluated only for erythromycin-resistant isolates. ^f^ Doxycycline has been tested in that study. ^g^ Minocycline has been tested in that study. ^h^ n.a.: not available.
